# Development of a Clinically Oriented Model to Predict Antitumor Effects after PD-1/PD-L1 Inhibitor Therapy

**DOI:** 10.1155/2022/9030782

**Published:** 2022-05-04

**Authors:** Xueping Wang, Zhonglian He, Wen Liu, Runkun Han, Huilan Li, Shuqin Dai, Lin Zhang, Minjie Mao

**Affiliations:** ^1^Department of Laboratory Medicine, State Key Laboratory of Oncology in South China, Collaborative Innovation Center for Cancer Medicine, Sun Yat-sen University Cancer Center, Guangzhou, China; ^2^Department of Information Section, State Key Laboratory of Oncology in South China, Collaborative Innovation Center for Cancer Medicine, Sun Yat-sen University Cancer Center, Guangzhou, China; ^3^Department of Laboratory Medicine, Eighth Affiliated Hospital of Sun Yat-sen University, Shenzhen, 518000 Guangdong, China

## Abstract

Immune checkpoint inhibitors (ICI) have created an advanced shift in the treatment of lung cancer (LC), but the existing biomarkers were not in clinical and widespread use. The purpose of this study was to develop a new nomogram with immune factors used for monitoring the response to ICI therapy. LC patients with PD-1/PD-L1 inhibitors treatment were included in this analysis. The immune biomarkers and clinicopathological characteristic values at baseline were used to estimate the tumor response. The nomogram was based on the factors that were determined by univariate and multivariate Cox hazard analysis. For internal validation, bootstrapping with 1000 resamples was used. The concordance index (*C*-index) and calibration curve were used to determine the predictive accuracy and discriminative ability of the nomogram. Overall survival (OS) was estimated using the Kaplan-Meier method. Patients with lung metastasis (*P* = 0.010), higher baseline neutrophil-lymphocyte ratio (NLR) level (*P* < 0.001), lower baseline lymphocyte-monocyte (LMR) (*P* = 0.019), and lower CD3^+^CD8^+^ T cell count (*P* = 0.009) were significantly related to the tumor response. The above biomarkers were contained into the nomogram. The calibration plot for the probability of OS showed an optimal agreement between the actual observation and prediction by nomogram at 3 or 5 years after therapy. The *C*-index of nomogram for OS prediction was 0.804 (95% CI: 0.739-0.869). Decision curve analysis demonstrated that the nomogram was clinically useful. Moreover, patients were divided into two distinct risk groups for OS by the nomogram: low-risk group (OS: 17.27 months, 95% CI: 14.75-19.78) and high-risk group (OS: 6.11 months, 95% CI: 3.57-8.65), respectively. A nomogram constructed with lung metastasis baseline NLR, LMR, and CD3^+^CD8^+^ T cell count could be used to monitor and predict clinical benefit and prognosis in lung cancer patients within ICI therapy.

## 1. Introduction

As the leading cause of cancer death in China and worldwide, the incidence of lung cancer has increased 11.6% and the death rate has increased 18.4% during the last year worldwide [1]. Although there are some therapy regimens, including combination of surgery, chemotherapy targeted therapy, most patients with advanced/metastatic lung cancer have poor survival and less than 5% of them survive beyond 5 years [2]. More effective and personalized therapies for lung cancer are urgently needed.

The application of immune checkpoint inhibitors (ICIs) has profoundly expanded the management in lung cancer, which revolutionized the treatment paradigms in advanced cancer [3]. Programmed cell death 1 (PD-1) is one of the most important inhibitory immune checkpoints, expressed on immune cells such as T cells and B cells. Currently, ICIs targeting the PD-1/PD-L1 pathway as monotherapy or combination treatment have reshaped the treatment of various cancers and become a standard treatment in lung cancer. The most commonly used antibodies included PD-1 antibodies and PD-L1 antibodies, such as nivolumab, pembrolizumab, and atezolizumab [4]. However, due to no appropriate indicators to select the proper population, only a small number of people patients (~20%) could benefit from the treatment of PD-1/PD-L1 antibody [5–7]. Given this, unselected usage of PD-1/PD-L1 inhibitor monotherapy in all the patients might lead to low rate of benefit, high costs, and even immune-related adverse events (irAE). Various efforts have been made to identify biomarkers of the response to anti-PD-1/PD-L1 immunotherapy. Expression of PD-L1 protein [8], effector T-cell gene expression [9], tumor mutational burden (TMB) measured in tumor tissue or peripheral blood [10, 11], Eastern Co-operative Oncology Group (ECOG) performance status, and routine hematological indicators such as peripheral blood cell counts and lactate dehydrogenase have been proposed to identify prognosis. Recent studies suggest that biomarker combination approaches may be the future of response prediction to ICI therapies than single biomarkers; however, there are few reports about it. The interaction between tumor cells, tumor microenvironment (TME), and immune system involved in the process of a normal cell transform to tumor cell. As a general concept, better understanding of the biological mechanisms in the clinical applicability of cancer immunotherapy by the different strategies is urgently needed, which is the constant feature in the journey of ICI therapy [12].

As a breakthrough in oncology, ICI has brought about an unimaginable improvement in the landscape of advanced-stage cancer treatment, such as lung cancer; thus, these treatments (anti-PD-1/PD-L1 immunotherapy) are now widely used in clinical practice. But the response to ICI is varied in patients with lung cancer; especially, some patients do not respond to anti-PD-1/PD-L1 immunotherapy accompanied by toxic side effects, which lead to discontinuation ICI and switch to the other therapies. The biomarkers are urgently needed for accurate estimation of the tumor responses and the clinical outcomes, which could have function in reducing useless and potentially harmful therapy, including social costs and pressure [13, 14].

A nomogram with statistical predictors has been accepted as reliable tools to predict patient clinical events, such as enrollment criteria, treatment stratification, and overall survival. However, currently, there are little such online tools available for prognosis of lung cancer with ICI therapy. The goal of this study was to identify prognostic factors from clinicopathologic variables and immune factors to develop a new prognostic model for LC patients with ICI therapy.

## 2. Materials and Methods

### 2.1. Population

125 LC patients with PD-1/PD-L1 ICI therapy between July 2011 and May 2019 at the Sun Yat-sen University Cancer Center were selected for this study. The exclusion criteria are as follows: (1) patients with other tumors; (2) patients who received PD-1/PD-L1 inhibitor therapy before in our hospital; and (3) patients without complete clinical data and follow-up information.

### 2.2. Intervention

All the advanced LCs patients, who received PD-1/PD-L1 inhibitor therapy (nivolumab, pembrolizumab, or atezolizumab) were included in our study.

Also, patients receiving PD-1/PD-L1 inhibitors and/or in combination with chemotherapy and anti-VEGF targeting therapy were also included in this analysis. Objective responses to ICI were determined by the response evaluation criteria in solid tumors (RECIST) version 1.1. Baseline assessments were done with a computed tomography scan (CT scan) of the chest and abdomen within 2 weeks before treatment, and then the oncological outcomes were assesses every 4 cycles of treatment. All patients provided written informed consent. The Institute Research Ethics Committee of the Sun Yat-Sen University Cancer Center, Guangzhou, China, approved this study.

### 2.3. Laboratory Measurements

Serum/plasma samples were collected at room temperature before treatment initiation (baseline visit) and then centrifuged at 3500 r/min for 10 min. All the biomarkers were detected using a commercially available flow cytometry assay according to the manufacturer's instructions, respectively. White blood cell count (WBC), neutrophil number, lymphocyte, and monocyte were estimated by Sysmex XN 2000 (Japan), while IL-6, IL-10, and T/B/NK cell count were estimated by BD FACS Canto II (USA). LMR is calculated by lymphocyte-monocyte ratio, and NLR is calculated by neutrophil-lymphocyte ratio. Basic patient information, including age, gender, preoperative histologic grade, and therapy method, was extracted from the Electronic Medical Record (EMR) system. All the biomarker values were obtained before PD-1/PD-L1 inhibitor therapy.

### 2.4. Outcome

The levels of all the biomarker detected after 4 cycles of PD-1/PD-L1 inhibitor were used in LC patients according to clinical guidelines.

Telephone follow-up was used to the patients who did not visit our hospital as scheduled to get the treatment information and living status (performed by The Medical Information Unit in our Cancer Center). The last follow-up time was June 2019. Overall survival (OS) was the outcome of our study, which is defined as the time from the start of ICI therapy to the date of the last follow-up or death.

### 2.5. Statistical Analysis

SPSS 16.0 (IBM, Chicago, IL, USA) and R software (version 3.1.4; http://www.Rproject.org) were used to the statistical analysis. Categorical variables were classified based on clinical findings. Continuous variables were transformed to categorical variables by the optimal cut-off points, defined by the reference range (IL-6, IL-10, CD3^+^, CD3^+^CD4^+^, CD3^+^C8^+^, CD4/CD8, CD19, and CD3-/CD16^+^/CD56^+^) and X-tile (WBC, NLR, and LMR). The risk factors were analyzed by univariate and multivariate regression analyses, which were selected to predict prognosis by the derivation of prediction models. And then a diagnostic model for LC patients with ICI therapy was developed relying on the risk factors from multivariable logistic analysis [15]. The nomogram could be used to predicting 3- and 5-year OS. Harrell's *C*-index was evaluated to quantify the discrimination performance of the nomogram. In brief, a nomogram with relatively good discrimination should have a *C*-index value greater than 0.75. Calibration was performed by observing and Kaplan-Meier estimating survival probability. The decision curve was plotted [16, 17]. The total points of each patient were calculated according to the nomogram, 2 groups of patients with high and low risk of prognosis (based on the total points) were delineated using maximally selected rank statistics as implemented in the maxstat package. The Kaplan-Meier method was used to plot survival curves, using the dichotomized risk group as a factor, finally, compared using the log-rank test. A two-tailed *P* value < 0.05 was considered statistically significant. The authenticity of this article has been validated by uploading the key raw data onto the Research Data Deposit public platform (http://www.researchdata.org.cn/), with the approval RDD number as RDDA2022927824.

## 3. Results

### 3.1. Clinicopathological Characteristics of LC Patients

A total of 125 patients treated with anti-PD-1 or anti-PD-L1 monotherapy were analyzed in our study. All the patients have estimated serum markers before ICI therapy, and with a baseline CT scan, the response was assessed after 4 cycles. The clinicopathological characteristics are described in Table 1. Among these patients, 37 (29.60%) and 88 (70.4%) patients exhibited death and survival, respectively. The age was 54.83 ± 10.68 (mean ± SD) years, and 72% males of patients were included. The histology of LC included adenocarcinoma (62.40%), squamous cell carcinoma (31.2%), small-cell carcinoma (2.40%), large cell carcinoma (0.08%), and sarcomatoid carcinoma (3.20%). Only 10 patients have mutation (8 patients have EGFR mutation, and 2 patients have ALK mutation). There were 97 patients having metastasis. All the variations of serum biomarkers before ICI therapy are summarized.

### 3.2. Correlation between Clinicopathological Characteristics, Serum Biomarkers, and OS

**Figure 1:**
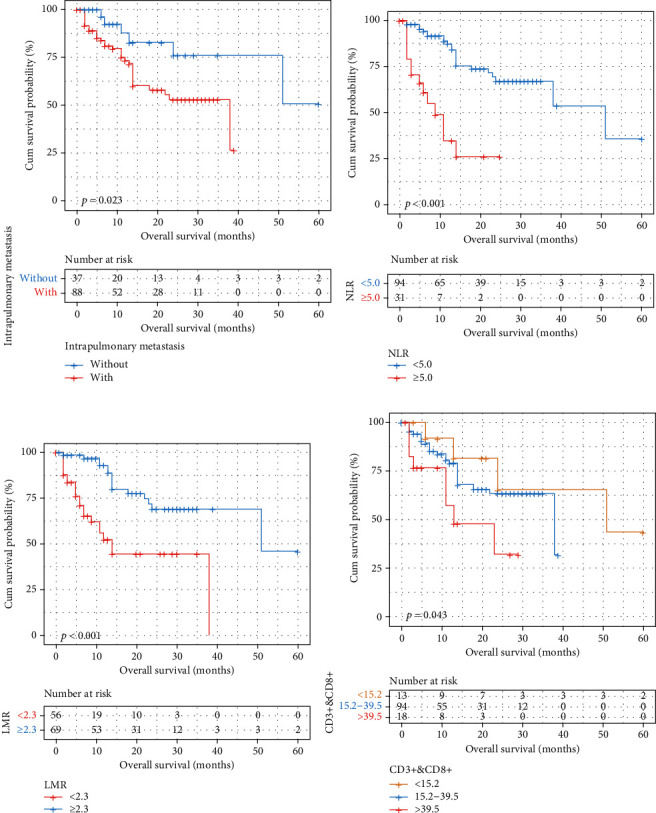
Overall survival (OS) stratified by prognostic nomogram in LC patients with ICI therapy. (a) LC patients with lung metastasis have short OS; (b) LC patients with high NLR level have short OS; (c) LC patients with low LMR level have short OS; (d) LC patients with high NLR level have short OS; (c) LC patients with high CD3^+^CD8^+^ T cell count have short OS.

Univariate and multivariate Cox proportional hazard regression analyses were used to estimate the relationship between all the biomarkers and OS (Table 2). A significant correlation between lung metastasis (*P* = 0.042), NLR (*P* < 0.001), LMR (*P* < 0.001), CD3^+^CD8^+^ T cell count (*P* = 0.026), and OS were determined by univariate analysis. Then, multivariate analyses were performed to identify factors selected by univariate analyses, and the above biomarkers were significant predictors of OS: lung metastasis (*P* = 0.010), NLR (*P* < 0.001), LMR (*P* < 0.019), CD3^+^CD8^+^ T cell count (*P* = 0.009) (Figure 1).

### 3.3. Development and Discrimination of the Prediction Model

**Figure 2:**
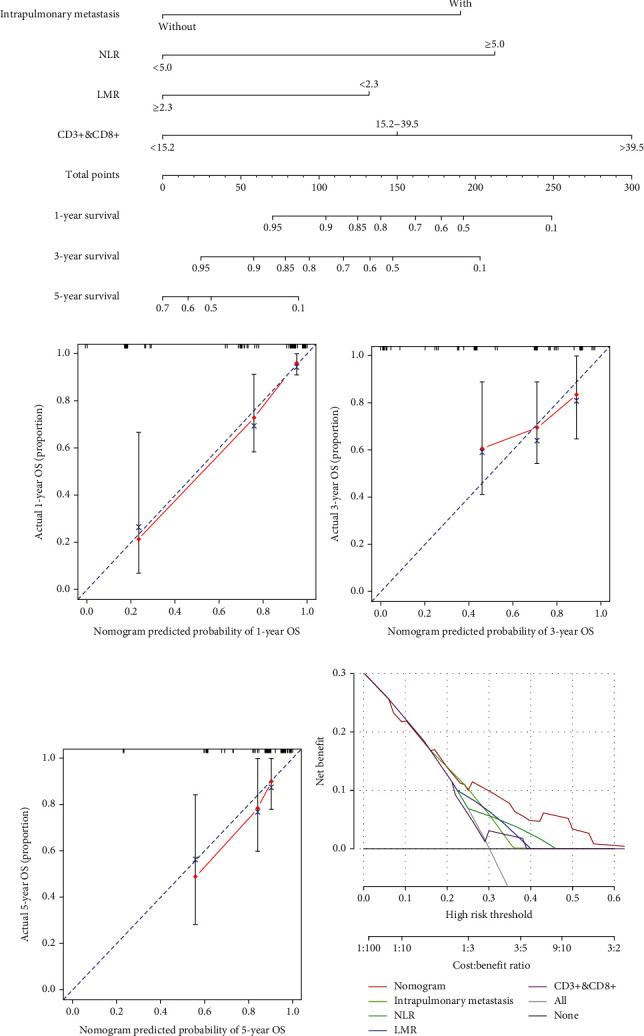
Nomogram (a) for the prediction of OS in LC patients with ICI therapy and its calibration plot of one-year (b). Three-year (c), five-year (d), and its decision curve analysis for OS (e).

A nomogram was constructed to predict the OS. On the basis of the serum factors identified by the Cox-analysis (Figure 2). *C*-index was performed to estimate the discrimination of nomogram. The predicted and observed Kaplan-Meier survival was compared for the evaluation of calibration, with the *y*-axes are observed survival calculated by the Kaplan-Meier method and the *x*-axes are actual survival estimated by the nomogram. The *C*-index for OS prediction was 0.804 (95% CI: 0.739-0.869). The calibration plot for the probability of OS showed an optimal agreement between the actual observation and prediction by nomogram at 1, 3, or 5 year after therapy (Figure 2).

### 3.4. Decision Curve Analysis

Figure 2(e) shows the decision curve analysis of the nomogram for OS, which presented that if the threshold probability of a patient is >10%, the developed nomogram in predicting OS is more benefit than none patients dead scheme or all patients dead scheme (Figure 2).

### 3.5. Relationship between Lung Metastasis, NLR, LMR, CD3^+^CD8^+^ T Cell Count, Nomogram, and OS

In the whole study, the mean OS was 14.77 months (95% CI: 12.59-16.95), and the mean OS in group of death was 11.32 months (95% CI: 7.91-14.74) compared with the median OS in group of death was 16.22 months (95% CI: 13.49-18.94). In particular, the Kaplan-Meier curves showed that patients with lung metastasis, high NLR, low LMR, and high CD3^+^CD8^+^ T cell count have the shorted OS (14.05 months vs.16.49 months, 6.00 months vs. 17.66 months, 9.43 months vs. 19.10 months, and 9.72 months vs. 14.48 vs. 23.85 months).

**Figure 3:**
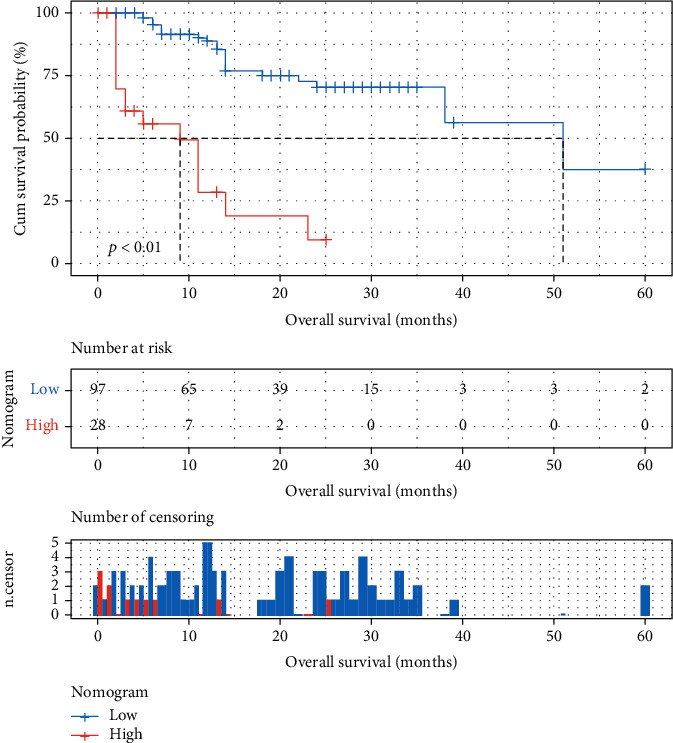
Kaplan-Meier survival analysis of LC patients on the nomogram treated with ICI.

Actually based on the nomogram, the first horizontal line represented the point values for each variable in vertical line; then all the corresponding points are summed to obtain the total points. Finally, from the total points we could have got the value of 1-, 3-, or 5-year OS probability. For example, the presence of lung metastases corresponds to 63 points, the presence of high NLR corresponds to 71 points, and the presence of high LMR corresponds to 0 points, while the high CD3^+^CD8^+^ T cell count corresponds to 99 points. The total point of 204 corresponds 1-, 3-, or 5-year OS of about 0.45 (45%) and 0.2 (20%), respectively. At last, the optimal cut-off value of total point was defined by X-tile as 160; thus, patients were subdivided into two groups: a low-risk group (with lower total point) and a high-risk group (with higher total point). In the cohort, the low-risk group had the longest OS 17.27 months (95% CI: 14.75-19.78), compared with the high-risk group 6.11 months (95% CI: 3.57-8.65). Furthermore, we drew the Kaplan–Meier curves, and the differences between these two groups were significant (*P* < 0.001) (Figure 3).

## 4. Discussion

As far as we know, this is the first study for developing a nomogram with serum immune indexes to measuring the response in lung cancer with anti-PD-1/PD-L1. For the nomogram construction, univariate and multivariate analyses have been used in our study, including clinical features, treatment history, and immune biomarkers. Interestingly, the multivariate analysis showed that the baseline of lung metastasis, NLR, LMR, and CD3^+^CD8^+^ T cell count were independent prognostic factors, which suggest that patients with lung metastasis, high NLR, low LMR, and high CD3^+^CD8^+^ T cells in baseline correlate with poor response in treatment of ICI, which suggest that LC patients with no lung metastasis, lower NLR, higher LMR, and higher CD3^+^CD8^+^ T cells show a better OS. The nomograms were combined with lung metastasis, NLR, LMR, and CD3^+^CD8^+^ T cell count. In predicting OS of lung cancer, the nomogram performed well with adequate discrimination in the primary cohort (*C*-index, 0.804 (95% CI: 0.739-0.869), and the calibration plot for the probability of OS showed an optimal agreement between the actual observation and prediction by nomogram at 3 or 5 year after therapy. Simultaneously, the decision curve showed that the nomogram does well in OS prediction in all range. Furthermore, based on the nomogram, patients were divided into two distinct risk groups for OS: the low-risk group had the better OS (17.27 months) (95% CI: 14.75-19.78), compared with the high-risk group (6.11 months) (95% CI: 3.57-8.65). Patients in the low-risk group have improved survival rates. Thus, the nomogram derived from prospectively collected data on 125 LC patients, composed of the lung metastasis and blood biomarkers in baseline, could be used to a more convenient biomarker for the prediction of OS and treatment strategy guidance for lung patients with PD-1/PD-L1 inhibitor therapy.

Tumor cells, immune cells, and inflammatory cytokines have closely interacted with each other in the TME, which act as immunosuppression by protecting tumor cells from being detected and eradicated by immunosurveillance. Within the TME, some other components might be associated with response with anti-PD-1/PD-L1 include immune T cells, monocytes, granulocyte, macrophages, and other inhibitory immune checkpoints and cytokines. Many studies have reported that the systemic immune-inflammation index (SII), based lymphocyte counts, neutrophil counts, and monocyte counts have been put forward and validated as powerful prognostic biomarkers in various tumors, such as lung cancer, hepatocellular carcinoma [18], and renal cell carcinoma [19]. In the advanced tumor stage, the inflammation-based cells interacted with tumor distant metastasis in a complicated and close model [20]. The tumor development has been reported to relate to abnormal counts and ratio of neutrophil, lymphocyte, and monocytes in the peripheral blood of cancer patients [21]. T cells play a key role in infection and cancer during the immune responses, through T cell receptor (TCR) binding to the peptide antigens of antigen-presenting cells (APCs) and/or abnormal cells. Also, T cells normally express inhibitory receptors on their surface, such as PD-1, which prevent T cell activation by binding to PD-L1. After the combination of PD-1 and pd-L1, the tumor-killing activity of T cells was suppressed and the T cell responsibility was downregulated. The functions of PD-1/PD-L1 binding in immune cells include the induction and maintenance of peripheral immune tolerance, protecting tissue from immune attack and dampening infectious immunity and tumor immunity [22]. Thus, tumors may escape the type of surveillance by PD-1/PD-L1. The development of antitumor effect depends not only on the interfering with PD1 signal transduction but also on the activating the cytotoxic T cells. Therefore, the CD3^+^CD8^+^ T cell count detection may be of more predictive value. In our study, we have consider the anatomical extent, the comprehensive systemic inflammatory biomarkers and immune biomarkers into our nomogram, which provide a more accurate prediction of the patient's prognosis after anti-PD-1/PD-L1.

There are also some limitations in our study. First, lacking multicenter research data, the nomogram was created based on single data source. Second, the PD-1/PD-L1 inhibitors of nivolumab and pembrolizumab were included in our study, which might have some response bias. Finally, since there are some inclusion and exclusion criteria of LC patients with PD-1/PD-L1 inhibitors, some patients have not been allowed to be included in our study, the validation cohort is needed in the future study. In addition, our study is a monoinstitutional report, which has all the clinical assessments, OS, and laboratory biomarkers performed consistently among all the patients before the treatment, and all the data were not missed. Despite these limitations, this nomogram based on the baseline of lung metastasis, NLR, LMR, and CD3^+^CD8^+^ T cell count represents a prognostic effect on LC patients with PD-1/PD-L1 inhibitors. We anticipate that this nomogram will stimulate ongoing research and multiple-center clinical research with large population will further improve and validate it.

## 5. Conclusions

We developed a nomogram with four available markers to predict survival during the treatment with PD-1/PD-L1 inhibitors for LC. This is the first study that has analyzed the correlation between the baseline of lung metastasis, NLR, LMR, and CD3^+^CD8^+^ T cell count and the tumor response, and the nomogram offers an easy-to-use tool for ICI prognosis.

## Figures and Tables

**Table 1:** Baseline clinical features of LC patients with ICI therapy.

Characteristics	Development cohort (*n* = 125)
	Mean ± SD/no. (%)
Age, year	54.83 ± 10.68
Sex	
Male/female	90 (72.00%)/35 (28.00%)
Status	
Death/survival	37 (29.60%)/88 (70.4%)
BMI	
<18.5; 18.5-24.99; ≥25.0	8 (6.40%)/95 (76.00%)/22 (17.60%)
Weight change	
Loss/stable/rise	28 (22.40%)/93 (74.40%)/4 (3.20%)
Smoking	
Never/past/current	64 (51.20%)/31 (24.80%)/39 (31.20%)
Alcohol	
Never/past/current	97 (77.60%)/8 (6.40%)/20 (16.00%)
Histology	
Adenocarcinoma/squamous cell carcinoma/small-cell carcinoma/large cell carcinoma/sarcomatoid carcinoma	78 (62.40%)/39 (31.2%)/3 (2.40%)/1 (0.08%)/4 (3.20%)
Targetable mutation	
EGFR/ALK/others	8 (6.40%)/2 (1.60%)/1 (0.80%)
Previous therapy	
Chemotherapy/radiation therapy/targeted therapy/operative/immunotherapy	45/11/9/15/2
Subsequent therapy	
Chemotherapy/radiation therapy/targeted therapy/operative/immunotherapy	99/23/40/11/5
ICI therapy	
PD-1/PD-L1	124/1
TNM stage	
I/II/III/IV	4/2/24/95
Tumor size	
T1/T2/T3/T4/Tx	14/28/37/40/6
Node stage	
N0/N1/N2/N3/Nx	9/8/51/51/6
Metastasis	
Yes/no	97/28
Metastasis location	
Lung/brain/liver/kidney/bone/adrenal glands/other	88/16/21/2/36/14/46
WBC	8.00 ± 4.06
NLR	4.45 ± 4.48
LMR	2.78 ± 1.46
IL6	
<10.3/≥10.3	75/50
IL10	
<4.91/≥4.91	119/6
CD3^+^	
<56.5/56.5-83.1/>83.1	23/91/11
CD3^+^CD4^+^	
<25.2/25.2-48.3/>48.3	31/79/15
CD3^+^CD8^+^	
<15.2/15.2-39.5/>39.5	13/94/18
CD4/CD8	
<0.5/0.5-2.6/>2.6	13/103/9
CD19^+^	
<5.2/5.2-16.3/>16.3	33/76/16
CD3-	
<6.2/6.2-34.8/>34.8	10/48/67

Data are presented as mean (SD) or *N* (%).

**Table 2:** Univariate and multivariate cox hazards analyses between clinical features and OS.

Characteristics	Univariate analysis		Multivariate analysis	
	HR (95% CI)	*P* value	HR (95% CI)	*P* value
Age, year	1.028 (0.538-1.965)	0.932		
Sex				
Male/female	0.788 (0.369-1.682)	0.538		
BMI				
<18.5; 18.5-24.99; ≥25.0	0.706 (0.349-1.431)	0.334		
Weight change				
Loss/stable/rise	1.538 (0.740-3.198)	0.249		
Smoking				
Never/past/current	0.984 (0.671-1.443)	0.936		
Alcohol				
Never/past/yes	0.897 (0.509-1.579)	0.705		
Histology				
Adenocarcinoma/squamous cell carcinoma/small-cell carcinoma/large cell carcinoma/Sarcomatoid carcinoma	1.377 (0.984-1.928)	0.062		
Targetable mutation				
EGFR	0.045 (0.000-14.460)	0.292		
ALK	0.045 (0.000-944.682)	0.541		
Previous therapy				
Chemotherapy	0.846 (0.414-1.728)	0.646		
Radiation therapy	0.562 (0.135-2.341)	0.428		
Targeted therapy	1.167 (0.357-3.813)	0.798		
Operative	1.703 (0.707-4.105)	0.235		
Immunotherapy	0.049 (0.000-1.157*E*7)	0.759		
Subsequent therapy				
Chemotherapy	0.475 (0.191-1.183)	0.811		
Radiation therapy	1.289 (0.609-2.729)	0.508		
Targeted therapy	0.775 (0.377-1.594)	0.488		
Operative	1.155 (0.428-3.120)	0.776		
Other immunotherapies	0.412 (0.055-3.086)	0.388		
TNM stage				
I/II/III/IV	1.540 (0.846-2.803)	0.158		
Tumor size				
T1/T2/T3/T4/Tx	1.083 (0.795-1.477)	0.612		
Node stage				
N0/N1/N2/N3/Nx	1.351 (0.926-1.969)	0.118		
Metastasis				
Yes/no	1.211 (0.517-2.839)	0.659		
Metastasis location				
Lung	2.811 (1.087-7.270)	0.033	3.645 (1.362-9.756)	0.010
Brain	0.909 (0.320-2.579)	0.858		
Liver	1.650 (0.749-3.635)	0.214		
Bone	0.478 (0.199-1.150)	0.099		
Kidney	2.898 (0.385-21.803)	0.301		
Other	0.760 (0.373-1.550)	0.451		
WBC				
<7.3/≥7.3	1.895 (0.979-3.668)	0.058		
NLR				
<5.0/≥5.0	5.275 (2.614-10.645)	<0.001	4.198 (1.911-9.221)	<0.001
LMR				
<2.3/≥2.3	0.268 (0.136-0.529)	<0.001	0.405 (0.190-0.862)	0.019
IL6				
<10.3/≥10.3	1.696 (0.877-3.281)	0.117		
IL10				
<4.91/≥4.91	0.606 (0.078-4.703)	0.632		
CD3^+^				
<56.5/56.5-83.1/>83.1	1.442 (0.793-2.632)	0.231		
CD3^+^CD4^+^				
<25.2/25.2-48.3/>48.3	0.955 (0.545-1.675)	0.873		
CD3^+^CD8^+^				
<15.2/15.2-39.5/>39.5	2.114 (1.091-4.095)	0.026	2.752 (1.287-5.883)	0.009
CD4/CD8				
<0.5/0.5-2.6/>2.6	1.300 (0.612-2.763)	0.495		
CD19^+^				
<5.2/5.2-16.3/>16.3	0.970 (0.561-1.677)	0.914		
CD3-/CD16^+^/CD56^+^				
<6.2/6.2-34.8/>34.8	1.442 (0.793-2.632)	0.231		

## Data Availability

The analyzed datasets generated during the study are available from the corresponding authors on reasonable request.
